# Hepatitis C virus NS4B induces the degradation of TRIF to inhibit TLR3-mediated interferon signaling pathway

**DOI:** 10.1371/journal.ppat.1007075

**Published:** 2018-05-21

**Authors:** Yisha Liang, Xuezhi Cao, Qiang Ding, Yanan Zhao, Zhenliang He, Jin Zhong

**Affiliations:** 1 CAS Key Laboratory of Molecular Virology and Immunology, Unit of Viral Hepatitis, Institut Pasteur of Shanghai, Chinese Academy of Sciences, Shanghai, China; 2 ShanghaiTech University, Shanghai, China; 3 University of Chinese Academy of Sciences, Beijing, China; Duke University Medical Center, UNITED STATES

## Abstract

Toll-like receptor 3 (TLR3) senses dsRNA intermediates produced during RNA virus replication to activate innate immune signaling pathways through adaptor protein TRIF. Many viruses have evolved strategies to block TLR3-mediated interferon signaling via targeting TRIF. Here we studied how hepatitis C virus (HCV) antagonizes the TLR3-mediated interferon signaling. We found that HCV-encoded NS4B protein inhibited TLR3-mediated interferon signaling by down-regulating TRIF protein level. Mechanism studies indicated that the downregulation of TRIF by NS4B was dependent on caspase8. NS4B transfection or HCV infection can activate caspase8 to promote TRIF degradation, leading to suppression of TLR3-mediated interferon signaling. Knockout of caspase8 can prevent TRIF degradation triggered by NS4B, thereby enhancing the TLR3-mediated interferon signaling activation in response to HCV infection. In conclusion, our work revealed a new mechanism for HCV to evade innate immune response by blocking the TLR3-mediated interferon signaling via NS4B-induced TRIF degradation.

## Author summary

We previously reported that hepatitis C virus (HCV) NS4B inhibits the RIG-I–like receptors (RLR)-mediated interferon signaling. Here we studied whether NS4B antagonizes the TLR3-mediated interferon signaling in the HCV infection. We found that NS4B protein inhibited TLR3-mediated interferon signaling by down-regulating TRIF protein level, a mechanism that is different from the inhibition of RIG-I-mediated signaling by NS4B. Further studies demonstrated that NS4B can activate caspase8 to promote TRIF degradation, leading to suppression of TLR3-mediated interferon signaling. Knockout of caspase8 can prevent the NS4B-induced TRIF degradation, and thus enhance the TLR3-mediated interferon signaling activation in response to HCV infection. In conclusion, our work revealed a new mechanism for HCV to inhibit the TLR3-mediated interferon signaling by NS4B-induced TRIF degradation.

## Introduction

Hepatitis C virus (HCV) is an enveloped, single-stranded RNA virus belonging to the *Flaviviridae* family. HCV has a 9.6-kb RNA genome and encodes a large polyprotein of over 3000 amino acids which is cleaved into structural proteins (core, E1 and E2) and nonstructural proteins (p7, NS2, NS3, NS4A, NS4B, NS5A and NS5B). HCV infects approximately 170 million people worldwide, about 80% of whom develop into persistent infection. Persistent HCV infection leads to severe liver diseases, such as liver cirrhosis and hepatocellular carcinoma [1]. No vaccine is available for preventing HCV infection. Interferon (IFN) plus ribavirin, the traditional therapy to treat chronic hepatitis C, is not always effective and has strong side effect. Recently developed direct-acting antiviral agents (DAA), including NS3 protease inhibitors, NS5A inhibitors and NS5B nucleotide inhibitors have greatly improved curing efficiency. However, the impact of these highly effective DAAs on global control of HCV infection remains to be seen in the long run as drug-resistant mutations, severe liver disease progression in DAA-cured patients and other newly emerging problems arise [2]. Therefore, HCV infection is still a big threat to human public health.

The innate immune system is the first line of host defense against invading viral pathogens, which is initiated by host pattern recognition receptors (PRRs) that recognize specific molecular structures known as pathogen-associated molecular pattern (PAMP) residing in invading pathogens or produced during pathogen replication [3]. There are three major classes of PRRs: Toll-like receptors (TLRs), RIG-I–like receptors (RLRs) and NOD-like receptors (NLRs) [4–6]. RLRs, cytosolic RNA helicases that recognize double-stranded RNA (dsRNA) or single-stranded RNA (ssRNA) with a triphosphate 5’ end, consist of three members RIG-I, MDA5 and LGP2 [7]. Previous study showed that the *in vitro* synthesized HCV 3’-untranslated regions (UTR) RNA can be recognized by RIG-I to trigger IFN response if transfected into hepatocytes, suggesting that the 3’-UTR RNA may contain HCV PAMP [8–10]. Using a hepatic cell line in which HCV infection induces strong IFN response, we recently demonstrated that MDA5 plays a predominant role in sensing HCV PAMP during HCV infection [11]. Furthermore, we showed that LGP2, another RLR member, is essential for HCV infection-induced IFN signaling, likely facilitating MDA5’s recognition of HCV PAMP [12]. To establish persistent infection, HCV has evolved multiple mechanisms to regulate and evade innate immunity [7, 13]. HCV NS3/4A serine protease can cleave MAVS, a critical adaptor protein in the RLR-mediated IFN activation, to block RLR-mediated signaling [14]. We and others showed that HCV NS4B protein can also inhibit RLR-mediated IFN activation by targeting STING, an adaptor protein facilitating IRF3 phosphorylation by TBK1 [15–17].

Despite extensive research in how HCV activates and evades RLR-mediated IFN signaling, less has been done for HCV evasion of TLR3-mediated IFN signaling. TLR3 is expressed mainly in early endosome and senses dsRNA which is produced during RNA virus replication [18, 19]. TIR-domain-containing adaptor protein including IFN-β (TRIF) is the sole adaptor protein of TLR3-mediated pathway, and is often targeted by viruses to evade host innate immunity [20–25]. Early studies showed that HCV NS3/4A serine protease targeted TRIF for its cleavage [24, 26]. Despite these studies, the molecular mechanism underlying HCV blocking TLR3 signaling pathway remains to be explored. In this study we investigated how HCV antagonizes the TLR3-mediated interferon signaling. We found that HCV-encoded NS4B inhibited TLR3-mediated interferon signaling by promoting TRIF protein degradation in a caspase8-dependent manner. Our work revealed a new mechanism for HCV to evade host innate immunity.

## Results

### NS4B inhibits TLR3-mediated interferon signaling

**Fig 1:**
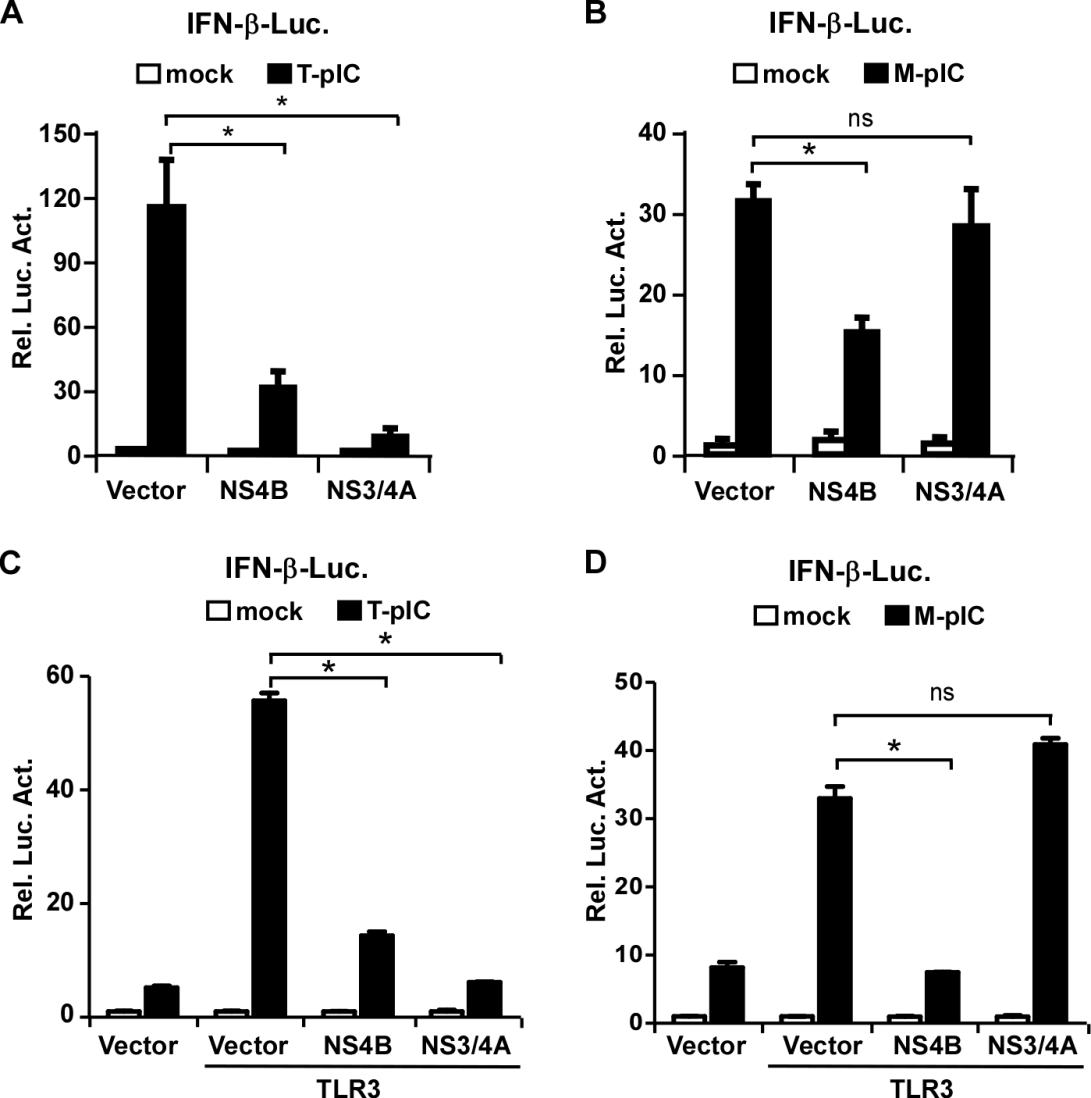
NS4B inhibits TLR3-mediated IFN signaling. **(A-B)** PH5CH8 cells were co-transfected with the IFN-β promoter-luciferease reporter plasmid and Flag-NS4B or NS3/4A expressing plasmid for 24 h, followed by either transfection with poly(I:C) (T-pIC) for 16 h **(A)** or treatment of poly(I:C) in culture medium (M-pIC) for 6 h **(B)**. (**C-D**) HEK293T cells were co-transfected with IFN-β promoter-luciferease reporter plasmid, TLR3 expressing plasmid and Flag-NS4B or NS3/4A expressing plasmid for 24 h, followed by poly(I:C) transfection (T-pIC) for 16 h **(C)** or poly(I:C) treatment (M-pIC) for 6 h **(D)**. The cells were harvested for luciferease assay. The luciferease activities were expressed as values relative to mock control. The error bars represent standard deviations from three independent experiments. One–way ANOVA was used for statistical analysis. ns, P>0.05; *P<0.05.

We and others previously showed that HCV encoded-NS4B protein can inhibit the RLR-mediated IFN signaling by targeting STING [15–17]. In this study we aimed to determine whether NS4B had any effects on the TLR3-mediated IFN signaling. A previous study showed that the addition of poly(I:C) to culture medium can activate the TLR3-mediated signaling in PH5CH8 cells, non-neoplastic hepatocytes transformed with large T antigen [27]. Therefore, we first used PH5CH8 cells to analyze the effect of NS4B on the TLR3-mediated signaling. Poly(I:C) was either added directly to the culture medium (M-pIC) or transfected (T-pIC) into PH5CH8 cells that were firstly transfected with a reporter plasmid expressing IFN-β promoter-driven luciferase and a plasmid expressing NS4B. In this setting, M-pIC and T-pIC would activate TLR3- and RLR-mediated interferon signaling respectively. In addition, HCV-encoded NS3/4A, known to cleave MAVS to block the RLR-mediated interferon signaling [28], was included as a control. As shown in Fig 1A and 1B, consistent with previous studies, both NS4B and NS3/4A decreased T-pIC-induced IFN-β response. However, NS4B but not NS3/4A decreased M-pIC-induced IFN-β response, suggesting that NS4B may have an inhibitory effect on the TLR3-mediated IFN signaling.

HEK293T cells lack TLR3 expression and thus are defective in the TLR3-mediated interferon signaling [29]. To investigate the effect of NS4B or NS3/4A on the TLR3-mediated interferon signaling in HEK293T cells, HEK293T cells were transfected with plasmids expressing TLR3, IFN-luciferase reporter and NS4B or NS3/4A, followed by poly(I:C) transfection (T-pIC) or treatment (M-pIC). Consistent with the observation in PH5CH8 cells, while both NS3/4A and NS4B decreased T-pIC-induced IFN-β response (Fig 1C), only NS4B suppressed M-pIC-induced IFN-β response in the TLR3-reconsitutted HEK293T cells (Fig 1D). Altogether, these results suggested that NS4B may suppress the TLR3-mediated IFN signaling.

### STING is not involved in the NS4B-inhibited TLR3 signaling pathway

**Fig 2:**
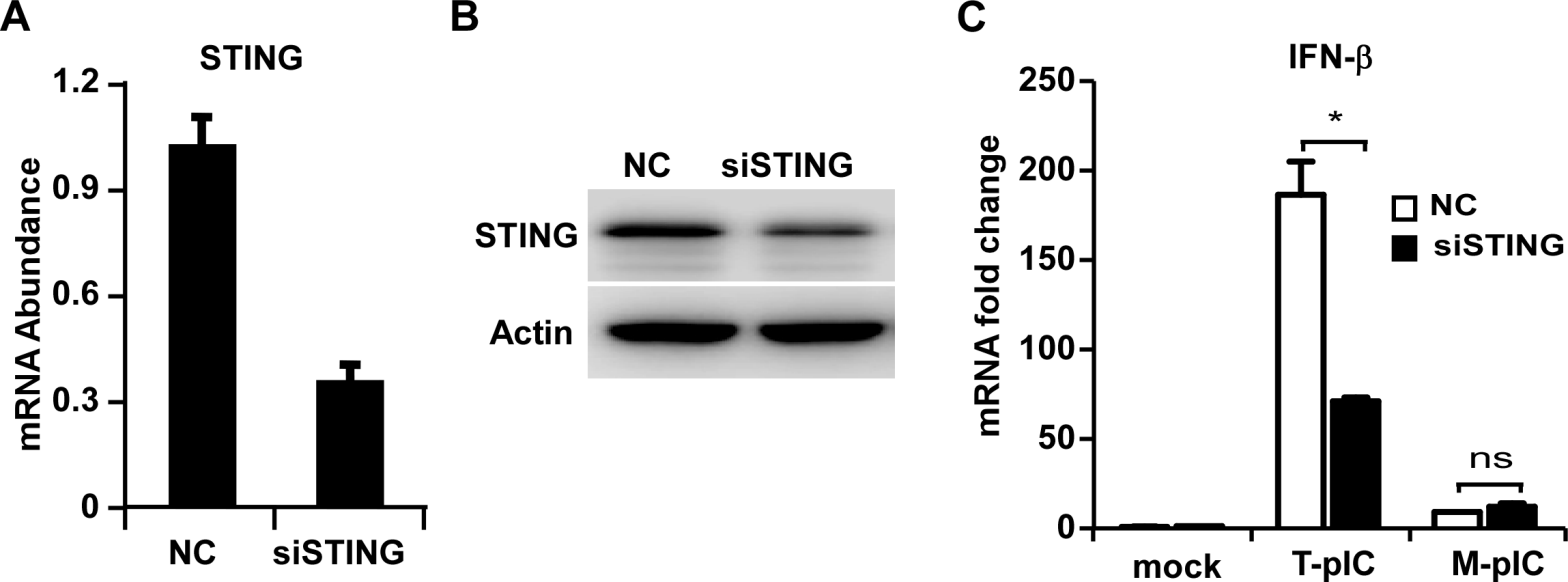
STING is not involved in the NS4B-inhibited TLR3 signaling pathway. PH5CH8 cells transfected with plasmids expressing STING-specific siRNA or unrelated siRNA (NC) for 48 h were either transfected with poly(I:C) (T-pIC) for 16 h or treated with poly(I:C) in culture medium (M-pIC) for 6 h. STING mRNA (**A**) and protein level (**B**) were analyzed by RT-qPCR or Western blot respectively. (**C**) The mRNA abundance of STING and IFN-β were analyzed by RT-qPCR, normalized against cellular Actin mRNA level and expressed as values relative to the mock control. The error bars represent standard deviations from three independent experiments. Student’s t test was used for statistical analysis. ns, P>0.05; *P<0.05.

STING is a transmembrane protein in the ER, and has a cytoplasmic domain that binds ligands to activate IFN signaling [30]. It has been reported that STING interacted with TRIF directly to trigger innate immune response to microbial infection [31]. We and others have shown that HCV NS4B protein can interact with STING to disrupt the RLR-mediated signaling [15–17]. To investigate whether the NS4B-STING interaction was also involved in NS4B suppression of the TLR3-mediated signaling, PH5CH8 cells were transfected with STING-specific siRNA followed by either poly(I:C) transfection (T-pIC) or treatment (M-pIC). Knockdown of STING expression was confirmed by RT-qPCR (Fig 2A) and Western blot (Fig 2B). As shown in Fig 2C, STING knockdown significantly decreased T-pIC-induced IFN-β response but had no effect on M-pIC-induced IFN-β response, suggesting that STING was not involved in the NS4B-inhibited TLR3 signaling pathway.

### NS4B decreases TRIF protein level

**Fig 3:**
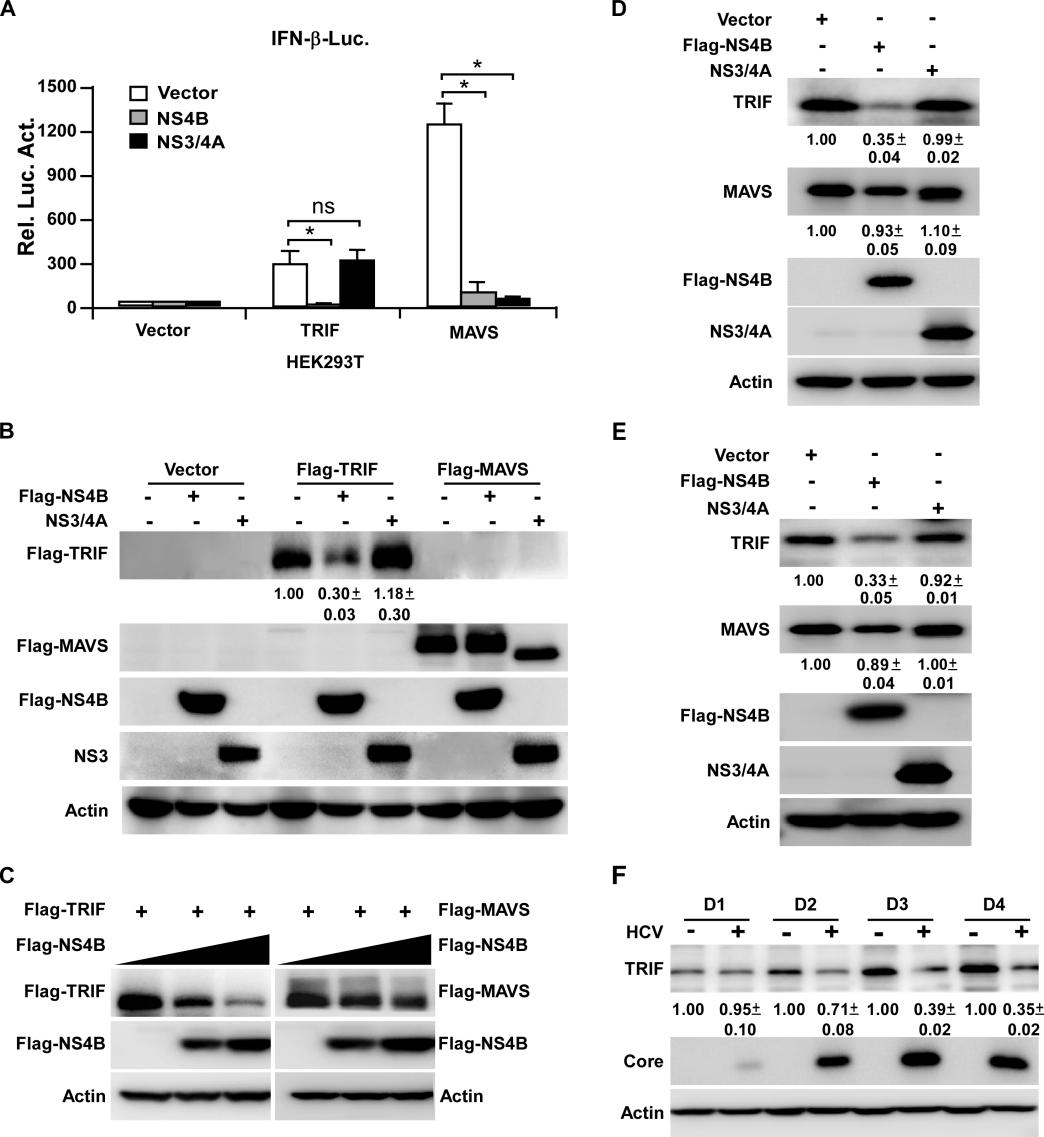
NS4B decreases TRIF protein level. (**A-B**) HEK293T cells were co-transfected with the IFN-β-luciferease reporter plasmid, Flag-NS4B or NS3/4A expressing plasmid, together with TRIF or MAVS expressing plasmid for 24 h. The cells were harvested for luciferase assay **(A)** or for analysis by immunoblotting with anti-Flag, anti-NS3 and anti-Actin antibodies **(B)**. The luciferease activities were expressed as values relative to mock control. The error bars represent standard deviations from three independent experiments. One-way ANOVA was used for statistical analysis. ns, P>0.05; *P<0.05. (**C**) HEK293T cells transfected with plasmid expressing TRIF or MAVS and increasing dose of plasmid expressing Flag-NS4B (0, 100 and 200 ng) for 48 h were analyzed by Western blot using anti-Flag, anti-Actin antibodies. (**D-E**) HEK293T cells **(D)** or PH5CH8 cells **(E)** transfected with Flag-NS4B or NS3/4A expressing plasmid for 48 h were analyzed by Western blot using anti-TRIF, anti-MAVS, anti-Flag, anti-NS3 and anti-Actin antibodies. (**F**) Huh7 cells infected with HCVcc (MOI = 5) for the indicated time points were analyzed by Western blot using anti-TRIF, anti-Core and anti-Actin antibodies. The protein levels were quantified by Image J from three independent experiments, normalized against internal Actin control and expressed as values relative to the mock infection controls.

Many viruses target TRIF to block TLR3 signaling [20–25], and early studies showed that HCV NS3/4A protease can cleave TRIF to shut down the TLR3-mediated signaling [24]. To examine a possibility that NS4B may target TRIF, we transfected HEK293T cells with plasmids expressing the IFN-β promoter-luciferase reporter, NS4B or NS3/4A, and TRIF or MAVS. As shown in Fig 3A, NS4B decreased both TRIF- and MAVS-mediated IFN-β response, while NS3/4A only decreased MAVS-mediated IFN-β response. Next we examined the effects of NS4B or NS3/4A on the protein levels of TRIF and MAVS. As shown in Fig 3B, NS4B had no obvious effect on MAVS protein expression as previously reported [15], but reduced TRIF protein level. In contrast, NS3/4A induced the cleavage of MAVS as previously reported [28], but had no effect on TRIF protein level. Furthermore, we transfected HEK293T cells with increasing dose of plasmids expressing NS4B and plasmids expressing TRIF or MAVS. As shown in Fig 3C, NS4B reduced the TRIF protein level in a dose-dependent manner but had no obvious effect on MAVS protein level.

Next we examined the effect of NS4B on endogenous TRIF protein expression. HEK293T (Fig 3D) and PH5CH8 (Fig 3E) cells were transfected with plasmids expressing NS4B or NS3/4A respectively, and the endogenous TRIF protein level was analyzed by Western blot. As shown in Fig 3D and 3E, NS4B decreased endogenous TRIF protein expression in both HEK293T and PH5CH8 cells.

Next we examined the TRIF protein expression in HCV-infected hepatocytes. Huh7 cells were infected with HCVcc at a multiplicity of infection (MOI) of 5, and the mRNA and protein levels of TRIF were determined at day 1, 2, 3 and 4 post-infection by RT-qRCR or Western blotting respectively. As shown in Fig 3F, HCV infection decreased endogenous TRIF protein level. In contrast, TRIF mRNA level was slightly increased in the HCV infected cells (S1A Fig). Altogether these results suggested that reduction of TRIF expression by NS4B likely occurred at a translational or post-translational level. We noted that the TRIF protein level increased over time in the mock infected cells (Fig 3F), probably resulting from the influences of cell density or nutrients in the culture medium on TRIF.

### Caspase8 is required for the NS4B-induced TRIF protein degradation

**Fig 4:**
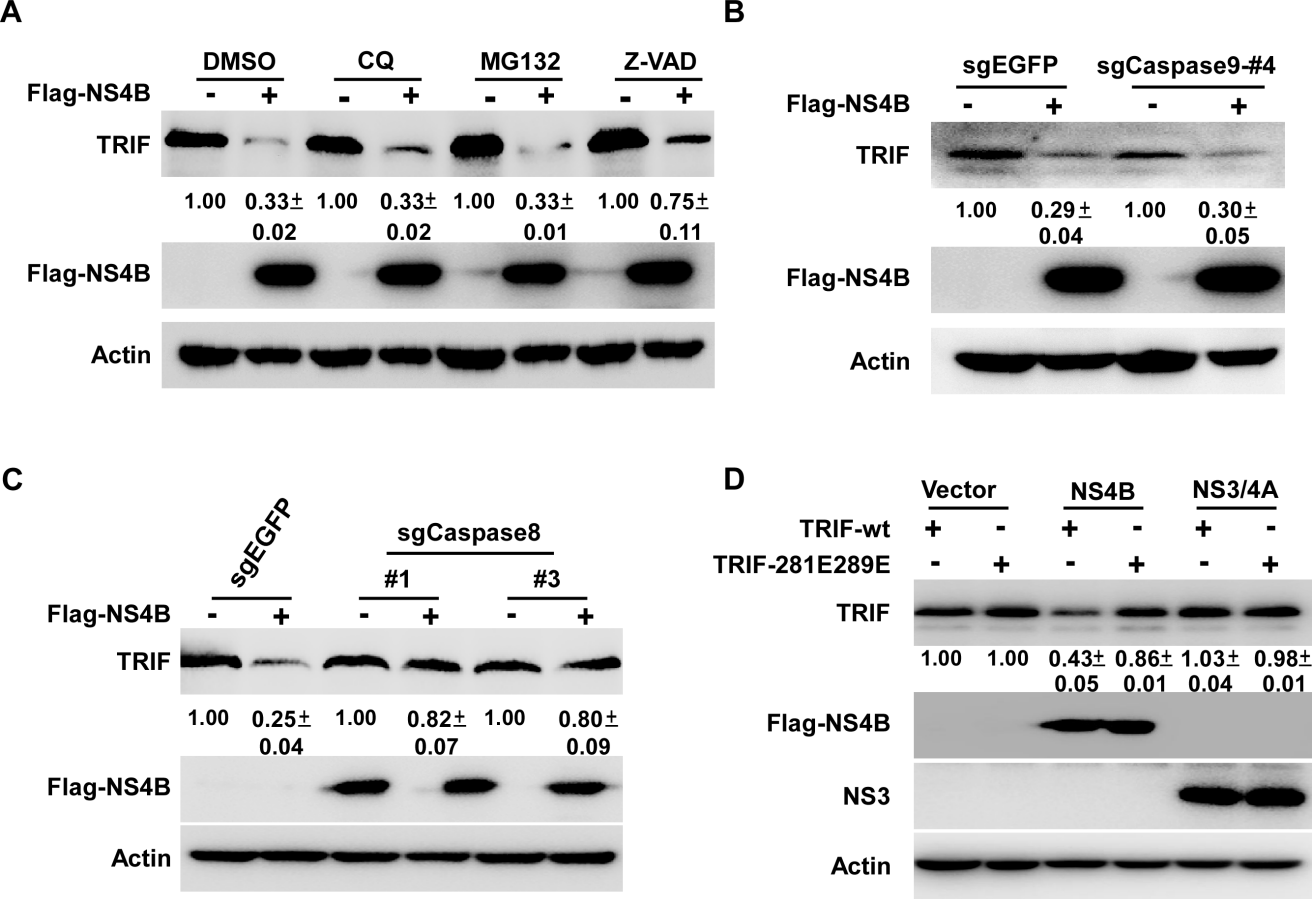
Caspase8 is required for the reduction of TRIF protein induced by NS4B. (**A**) HEK293T cells transfected with the plasmid expressing Flag-NS4B for 6 h were treated with DMSO control, chloroquine (20 μM) or Z-VAD-FMK (20 μM) for another 42 h, MG132 (5 μM) for 6 h. The cells were analyzed by immunoblotting with anti-TRIF, anti-Flag and anti-Actin antibodies. (**B-C**) HEK293T cells transduced with sgRNAs targeting caspase9, caspase8 or control (EGFP) were transfected with plasmid expressing Flag-NS4B. The cells were analyzed by immunoblotting with anti-TRIF, anti-Flag and anti-Actin antibodies. (**D**) HEK293T cells transfected with plasmids expressing Flag-NS4B or NS3/4A together with plasmids expressing TRIF-wt or TRIF-281E289E for 48 h were analyzed by immunoblotting with anti-TRIF, anti-Flag, anti-NS3 and anti-Actin antibodies. The TRIF protein levels were quantified by Image J from two independent experiments, normalized against internal Actin control and expressed as values relative to the mock infection controls.

Next we investigated potential mechanisms involved in the NS4B-induced TRIF protein degradation. HEK293T cells transfected with the NS4B-expressing plasmid were treated with different inhibitors of protein degradation pathways, including chloroquine (lysosome inhibitor), MG132 (proteasome inhibitor) and Z-VAD-FMK (caspases inhibitor), and the TRIF protein level was determined by Western blot. As shown in Fig 4A, the NS4B-triggered TRIF protein degradation was partially rescued by Z-VAD-FMK but not by chloroquine or MG132, suggesting that the NS4B-induced reduction of TRIF protein level may involve caspase-mediated protein degradation.

TRIF has been shown to undergo caspase8 or caspase9-dependent cleavage [32]. To determine whether caspase8 or caspase9 played a role in the reduction of TRIF protein level induced by NS4B, we constructed caspase8- or caspase9-knockout HEK293T cells using CRISPR-Cas9 technology (S2 Fig). HEK293T cells stably expressing caspase9-sgRNA (#4) or caspase8-sgRNAs (#1 and #3) displayed decent efficiency in reducing caspase9 or caspase8 expression, and thus were chosen for the further study (S2 Fig). These caspase9- or caspase8-knockout cells as well as control HEK293T cells expressing sgEGFP were transfected with plasmids expressing Flag-tagged NS4B, and the endogenous TRIF expression was determined by Western blot. As shown in Fig 4B, caspase9 knockout had no apparent effect on the NS4B-triggered TRIF protein degradation. In contrast, caspase8 knockout rescued the NS4B-triggered TRIF protein degradation (Fig 4C), suggesting that caspase8 was critical for the TRIF protein degradation induced by NS4B.

It was reported that two amino acid residues (281D and 289D) of TRIF are critical for the FasL-triggered TRIF cleavage by caspase8 [32], therefore we next examined the effect of point mutation of these two residues on NS4B-triggered TRIF degradation. Aspartic acid was mutated to glutamic acid at these two residues in TRIF (TRIF-281E289E). Next, plasmids expressing TRIF-wt or TRIF-281E289E were co-transfected with plasmids expressing NS4B or NS3/4A into HEK293T cells, and TRIF expression level was determined by Western blot. As shown in Fig 4D, while the wild-type TRIF protein expression was reduced by NS4B, the mutant TRIF protein expression level remained unchanged, suggesting that 281D and 289D are important for NS4B-triggered TRIF degradation.

### HCV infection induces the IFN signaling in the TLR3-reconstituted Huh7 cells

Although primary human hepatocytes (PHH) express TLR3, hepatoma-derived Huh7 cells, the sole human hepatic cell line that supports efficient HCV infection i*n vitro* and has been widely used for studying HCV infection and replication in cell culture, do not express a detectable level of TLR3 and thus are defective in the TLR3-mediated IFN signaling [27]. To study how NS4B-triggered TRIF degradation affects the TLR3-mediated signaling in response to HCV infection, we constructed Huh7 cells stably expressing TLR3 by lentiviral transduction. The expression of TLR3 was verified by Western blot (S3A Fig). Huh7-TLR3 cells were treated by poly(I:C) in culture medium (M-pIC), and the IFN-β and MxA mRNA levels were determined by RT-qPCR. As shown in S3B and S3C Fig, M-pIC induced IFN-β and MxA production in Huh7-TLR3 cells, but not in Huh7 cells, suggesting that the TLR3-mediated IFN signaling was restored in Huh7-TLR3 cells.

**Fig 5:**
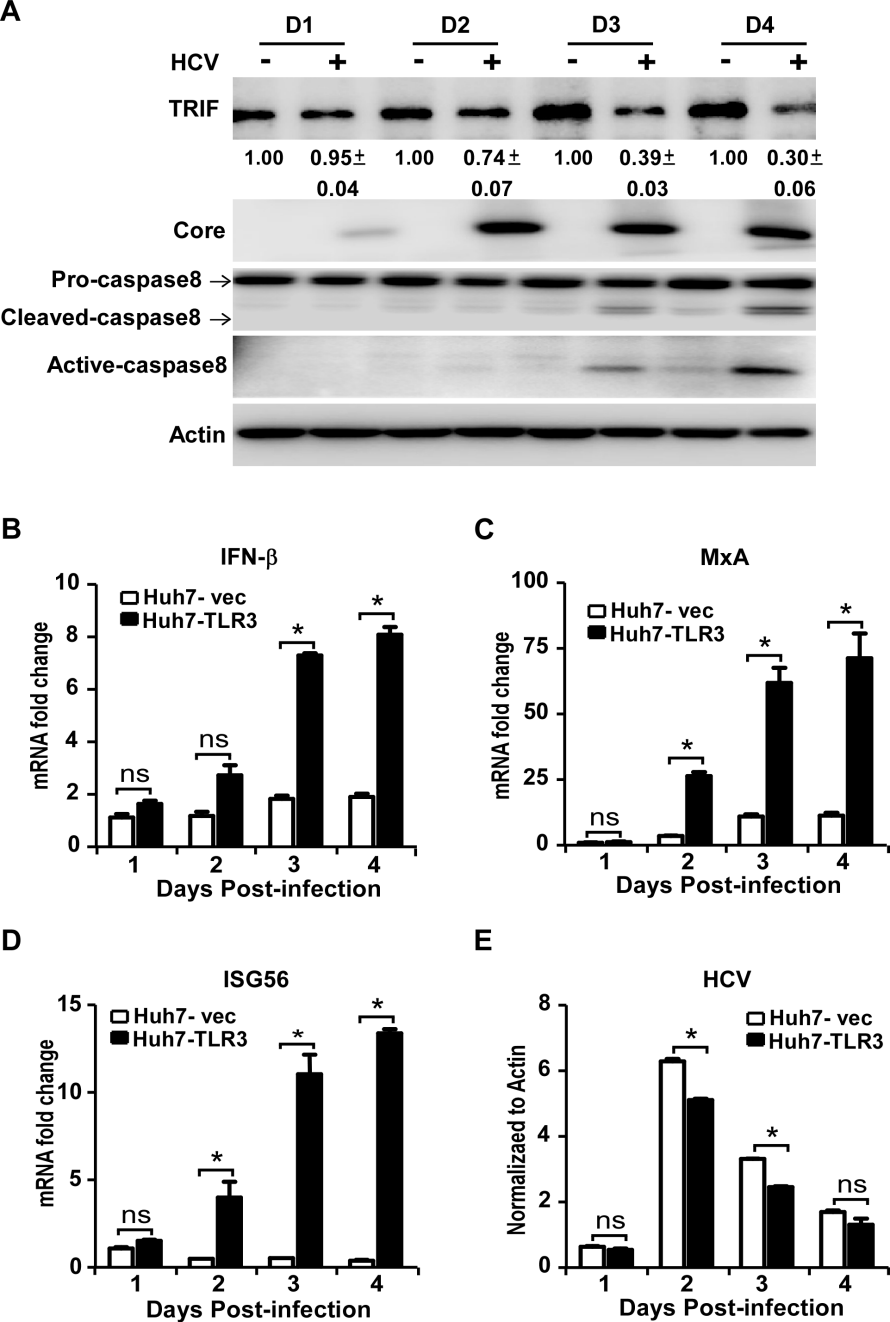
HCV infection activates caspase8 and reduces TRIF protein level in Huh7-TLR3 cells. Huh7-TLR3 cells were infected by HCVcc (MOI = 5) for the indicated time points, and analyzed by immunoblotting using anti-TRIF, anti-Caspase8, anti-Core and anti-Actin antibodies **(A)** or by RT-qPCR to detect the mRNA abundance of IFN-β **(B)**, MxA **(C)**, ISG56 **(D)** and HCV **(E)**. The TRIF protein level was quantified by Image J, normalized against internal Actin control and expressed as values relative to the mock infection controls from two independent experiments. The IFN-β, MxA and ISG56 mRNAs were normalized against cellular Actin mRNA level and expressed as values relative to the mock infection control. HCV RNA was expressed as values relative to the Actin mRNA level. The error bars represent standard deviations from three independent experiments. Student’s t test was used for statistical analysis. ns, P>0.05; *P<0.05.

Next we infected Huh7-TLR3 cells with HCVcc at MOI of 5, and determined the endogenous TRIF protein level and caspase8 activation by Western blot. Consistent with the observations in HCV-infected Huh7 cells (Fig 3F), HCV infection decreased endogenous TRIF protein level in Huh7-TLR3 cells (Fig 5A). In addition, HCV infection resulted in cleavage of pro-caspase8, a hallmark of caspase8 activation (Fig 5A). To investigate whether HCV infection induces IFN response in Huh7-TLR3 cells, we infected Huh7-TLR3 and control Huh7-vec cells with HCVcc at an MOI of 5, and determined IFN-β and ISGs mRNA levels by RT-qPCR. As shown in Fig 5B–5E, the presence of TLR3 significantly augmented the HCV infection-induced IFN signaling in Huh7-TLR3 cells. These results suggested that HCV infection still activates the TLR3-mediated IFN signaling while reducing the critical adaptor TRIF protein level in Huh7-TLR3 cells, possibly due to incomplete inhibition of TRIF by NS4B.

**Fig 6:**
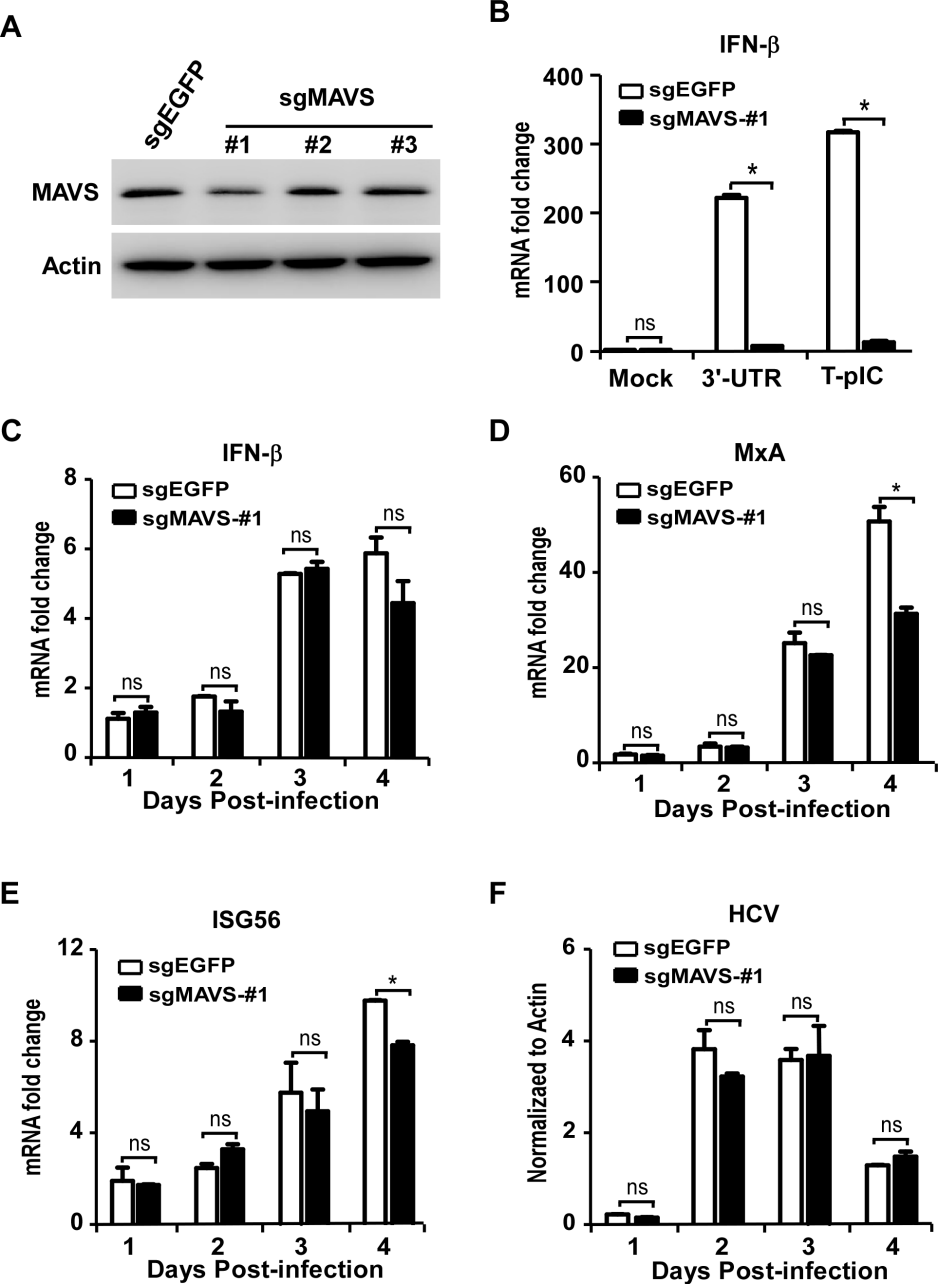
RLR signaling is dispensable for TLR3-mediated interferon signaling during HCV infection. **(A)** Huh7-TLR3 cells transduced with sgRNAs targeting MAVS or control EGFP were analyzed by immunoblotting using anti-TRIF and anti-Actin. **(B)** Huh7-TLR3-sgMAVS-#1 cells and control cells were transfected with 3’-UTR or poly(I:C) for 16 h, and analyzed by RT-qPCR to detect the mRNA abundance of IFN-β. **(C-F)** Huh7-TLR3-sgMAVS-#1 cells as well as control cells were infected by HCVcc (MOI = 5) for the indicated time points. The cells were analyzed by RT-qPCR to detect the mRNA abundance of IFN-β **(C)**, MxA **(D)**, ISG56 **(E)** and HCV **(F)**. The IFN-β, MxA and ISG56 mRNAs were normalized against cellular Actin mRNA level and expressed as values relative to the mock infection control. HCV RNA was expressed as values relative to the Actin mRNA level. The error bars represent standard deviations from three independent experiments. Student’s t test was used for statistical analysis. ns, P>0.05; *P<0.05.

Next we assessed potential effects of the RLR-mediated signaling on TLR3-mediated IFN signaling in HCV-infected Huh7-TLR3 cells. To do so, we knocked out the expression of MAVS, a critical adaptor protein in the RLR-mediated IFN signaling using the CRISPR-Cas9 technology (Fig 6A). The knockout of MAVS significantly diminished the IFN-β expression triggered by transfection of HCV 3’-UTR RNA or poly(I:C) (T-pIC), two ligands known to activate RIG-I- or MDA5-mediated IFN signaling respectively [11] (Fig 6B), suggesting that the RLR signaling was impaired in this MAVS-knockout cell. Nevertheless, this cell line remains fully responsive to the M-pIC-induced TLR3 signaling (S4 Fig). We then infected Huh7-TLR3-sgMAVS-#1 cells and control Huh7-TLR3-sgEGFP cells with HCVcc at an MOI of 5. The IFN-β and ISGs mRNA levels were determined by RT-qPCR. As shown in Fig 6C, RLR signaling blockade by knocking out the adaptor protein MAVS had no significant effect on the IFN-β induction in the Huh7-TLR3 cells, suggesting that the activation of the TLR3-mediated IFN signaling during HCV infection does not involve the RLR signaling. There was a slight decrease of MxA and ISG56 mRNA in the MAVS-knockout cells on day 4 post-infection (Fig 6D and 6E), which may result from amplification of IFN signaling contributed by the RLR signaling at late time points given that both RIG-I and MDA5 are themselves interferon-stimulated genes and can be up-regulated by the TLR3-mediated signaling.

### Caspase8 knockout blocks TRIF degradation and promotes TLR3-mediated IFN signaling during HCV infection

**Fig 7:**
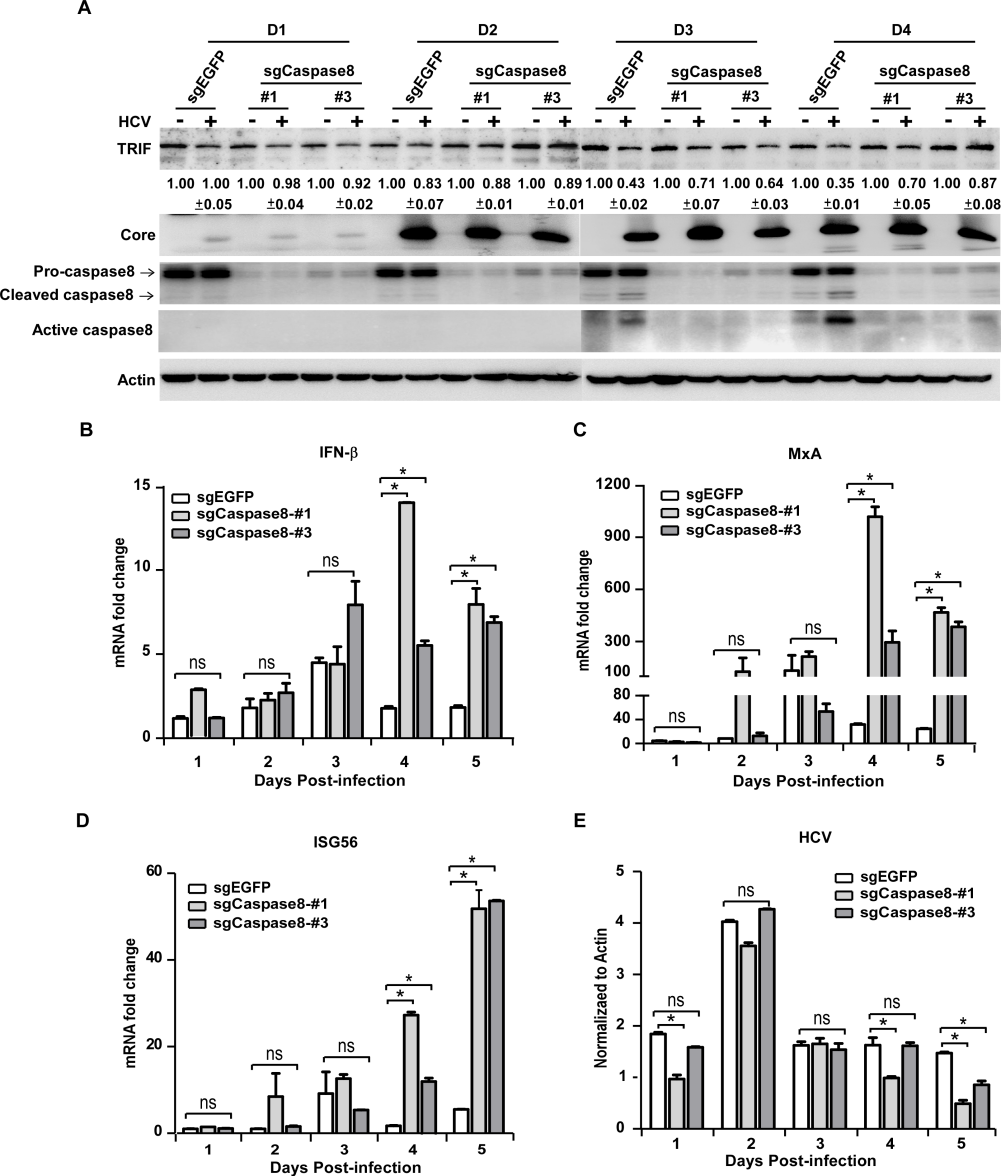
Caspase8 knockout blocks TRIF degradation and promotes TLR3-mediated IFN signaling during HCV infection. Huh7-TLR3 cells transduced with sgRNAs targeting caspase8 or control EGFP were infected by HCVcc (MOI = 5) for the indicated time points. The cells were analyzed by immunoblotting using anti-TRIF, anti-Caspase8, anti-Core and anti-Actin antibodies **(A)** or by RT-qPCR to detect the mRNA abundance of IFN-β **(B)**, MxA **(C)**, ISG56 **(D)** and HCV **(E)**. The TRIF protein level was quantified by Image J, normalized against internal Actin control and expressed as values relative to the mock infection controls from two independent experiments. The IFN-β, MxA and ISG56 mRNAs were normalized against cellular Actin mRNA level and expressed as values relative to the mock infection control. HCV RNA was expressed as values relative to the Actin mRNA level. The error bars represent standard deviations from three independent experiments. One-way ANOVA was used for statistical analysis. ns, P>0.05; *P<0.05.

To assess the contribution of NS4B/caspase8-mediated TRIF degradation to suppression of TLR3 signaling during HCV infection, we knocked down caspase8 expression in Huh7-TLR3 cells. Huh7-TLR3 cells were transduced with lentivirus expressing caspase8-specific sgRNAs (#1 and #3) that efficiently reduced caspase8 expression in HEK293T cells (Figs 4C and S2B). The knockout of caspase8 in the transduced Huh7-TLR3 cells was verified by Western blot (S5 Fig). These cells and control Huh7-TLR3 cells (sgEGFP) were infected with HCVcc. The endogenous TRIF protein level and caspase8 activation were analyzed by Western blot, and IFN-β and ISGs mRNA levels were determined by RT-qPCR. As shown in Fig 7A, HCV infection activated caspase8 and reduced TRIF protein level in the control cells, but had much less effect on TRIF protein level in the caspase8-knockout cells. Importantly, restoration of TRIF protein level in the caspase8-knockout cells was accompanied by the enhanced IFN-β, MxA and ISG56 mRNA levels (Fig 7B–7D). HCV mRNA levels were comparable among the three cells in the first 3 days after infection, but were lower in the caspase8-knockout cells on day 4 and 5 post-infection (Fig 7E), possibly due to more active antiviral IFN signaling in these cells.

To further confirm the role of caspase8 in the NS4B-mediated TRIF degradation and HCV infection-induced IFN signaling, we subcloned Huh7-TLR3-sgcaspase8-#1 cells and obtained a single clone of caspase8-knockout cell (Huh7-TLR3-sgcaspase8-#1-c4). The caspase8 knockout was verified by genomic sequencing and Western blot (S6A Fig). We then infected Huh7-TLR3-sgcaspase8-#1-c4 and control cells with HCVcc at an MOI of 5, and determined the TRIF expression by Western blot as well as the IFN-β and ISGs mRNA levels by RT-qPCR. Consistent with the observations in Fig 7, caspase8 knockout restored TRIF protein levels (S6B Fig) and enhanced IFN signaling in the HCV-infected Huh7-TLR3 cells (S6C–S6F Fig). Altogether, these results suggested that NS4B promoted caspase8-dependent TRIF degradation to suppress the TLR3-mediated IFN signaling during HCV infection.

## Discussion

To establish chronic infection, HCV has evolved multiple strategies to counteract IFN signaling. It has been demonstrated that HCV-encoded NS3/4A serine protease cleaves MAVS and TRIF to shut down the IFN signaling mediated by RLRs and TLR3 [24, 28]. Growing evidence showed that HCV employs additional strategies to disrupt the RLR- and TLR3-mediated IFN signaling. We and others previously reported that HCV NS4B can block RLR-mediated interferon signaling by targeting STING [15–17]. In this study, we provided several lines of evidence to demonstrate that NS4B can also disrupt the TLR3-mediated signaling by targeting the adaptor protein TRIF for its degradation. First, NS4B transfection blocks IFN signaling activated by extracellular poly(I:C), known to be recognized by TLR3 (Fig 1). Second, the disruption of TLR3-mediated signaling by NS4B was associated with the reduction of TRIF at the protein level (Fig 3A–3E), but not at the mRNA level (S1A Fig). Third, the NS4B-triggered TRIF protein degradation and blockade of TLR3-mediated IFN signaling were recapitulated in HCV infected cells (Figs 3F, 5A and 7), suggesting that interference of the TLR3-mediated IFN signaling by NS4B indeed takes place in the context of HCV infection.

The RLR-mediated IFN signaling in HCV-infected hepatocytes has been extensively studied, but less has been done so for the TLR3-mediated IFN signaling during HCV infection. We showed that HCV infection does trigger the TLR3-mediated IFN signaling (Fig 5). The magnitude of IFN activation in the HCV-infected Huh7-TLR3 cells is relatively low (less than 10-fold induction), likely due to the suppression of NS4B. When this NS4B-mediated suppression is relieved by knocking out caspase8, the IFN induction level can be further enhanced (Figs 7 and S6). These data demonstrated the importance of the TLR3 signaling in innate immune response against HCV infection and also highlighted the necessity for HCV to control this innate immune pathway. Furthermore, we showed that the RLR signaling blockade had no significant effect on the TLR3-mediated IFN activation during HCV infection (Fig 6). In addition, we showed that the enhancement of TLR3-mediated IFN signaling by knocking out caspase8 and preventing TRIF degradation was not affected by the RLR signaling blockade (S7 Fig). These results suggest that the TLR3 and the RLR signalings are likely two independent host innate immune responses to HCV infection, which the virus must find ways to evade simultaneously.

We explored potential molecular mechanisms underlying down-regulation of TRIF protein level by NS4B. Unlike NS3/4A, NS4B does not have an enzymatic activity to cleave TRIF. Co-immunoprecipitation assays showed that NS4B did not directly interact with TRIF. Our results showed that NS4B-triggered TRIF degradation was caspase-dependent. Z-VAD-FMK, a pan-caspase inhibitor partially restored the TRIF protein expression in the presence of NS4B (Fig 4A). Our results showed that knockout of caspase8 but not caspase9 blocked the NS4B-induced TRIF degradation (Figs 4C and 7A) and enhanced IFN signaling in response to HCV infection (Fig 7). Furthermore, we showed that HCV infection indeed activates caspase8 in the cells particularly at late time points of infection, coincide with the onset of TRIF degradation and reduction in the interferon signaling (Figs 5 and 7). Two amino acid residues 281D and 289D in TRIF that were previously reported to be critical for the FasL-induced TRIF cleavage by caspase8 [32] seemed to be also important for NS4B-triggered TRIF degradation (Fig 4D), suggesting that NS4B-triggered TRIF degradation may share the similar molecular mechanism with FasL-induced TRIF cleavage by caspase8. NS4B is an ER membrane-associated protein and can induce morphological changes of ER membrane during active HCV genome replication, leading to ER stress [33, 34]. It has been reported that ER stress induces FADD oligomerization, which in turn interacts and activates caspase8 through its death effector domain (DED) [35]. Therefore, it is conceivable to speculate that NS4B may cause ER stress to activate caspase8. More research will be needed to test this hypothesis and to understand how the caspase8 activation eventually leads to the TRIF degradation.

Previous studies reported that the activation of TLR3 signaling also leads to apoptosis in a manner that requires the involvement of TRIF [36, 37]. Our finding that NS4B promotes the TRIF degradation raises a possibility that NS4B may also counter apoptosis of HCV-infected hepatocytes, which may contribute to hepatocyte proliferation and liver regeneration, an important prerequisite for development of hepatocellular carcinoma. More studies will be needed to evaluate the potential role of NS4B in antagonizing apoptosis of host hepatocytes.

Previous studies demonstrated that TRIF was targeted by HCV-encoded NS3/4A protease for its proteolysis in cell free system and in HEK293 and Huh7 cells [24, 26]. Interestingly, our results showed that HCV NS3/4A serine protease did not induce the cleavage or degradation of TRIF in HEK293T and PH5CH8 cells. Of notes, our results were consistent with some other groups’ finding that NS3/4A is incapable of cleaving TRIF in PH5CH8 cells and human primary hepatocytes [38–40]. This difference may result from possible different subcellular localization of TRIF and/or NS3/4A in the different cells as well as technical difficulties in detecting low level of TRIF proteins as suggested in other literature [41]. In addition, NS3/4A may act together with NS4B to degrade TRIF in the context of HCV infection. More research will be needed to address this issue.

In summary, we found that HCV NS4B protein induces caspase8-dependent TRIF degradation to block TLR3 signaling. Our work revealed a new strategy for HCV to evade innate immune response and should help understand molecular mechanisms underlying persistent HCV infection.

## Materials and methods

### Cell culture and virus preparation

HEK293T, Huh7 and their derivative cells were maintained in complete Dulbecco’s modified Eagle’s medium (DMEM) (Invitrogen, Carlsbad, CA, USA) supplemented with 10% fetal bovine serum, 10 mM HEPES, 2 mM L-glutamine, 100 U of penicillin/ml, and 100 mg of streptomycin/ml. PH5CH8 cells were maintained in DMEM/F12 (1:1) supplemented with 2 mM L-glutamine, 100 ng/ml epidermoid growth factor (Toyobo, Osaka Japan), 10 μg/ml insulin (Sigma-Aldrich, St. Louis, MO, USA), 5 μg/ml linoleic acid (Sigma-Aldrich), 106 nM hydrocortisone (Sigma-Aldrich), 107 nM selenium (Sigma-Aldrich), 5μg/ml transferrin (Sigma-Aldrich), 100 ng/ml prolactin (Sigma-Aldrich), and 2% fetal bovine serum. All cells were cultured in humidified air containing 5% CO_2_ at 37°C. HCVcc preparation was as previously described [42].

### Luciferase reporter assay

8 x 10^4^ PH5CH8 or HEK293T cells seeded in 48-well plates overnight were transfected with 20 ng/well plasmid expressing IFN-β-Luciferase reporter [11], 20 ng/well of plasmid expressing CMV promoter-driven Renilla luciferase and 300 ng/well plasmid expressing NS4B or NS3/4A. One day after transfection, the cells were either transfected with 400 ng/well poly(I:C) (Invivogen, San Diego, CA, USA) for 16 h or treated with 50 μg/ml poly(I:C) in culture medium for 6 h. Cell lysates were assayed for the luciferase activity using the Dual-Luciferase Reporter Assay System (Promega).

### RNA isolation and quantitative RT-PCR (RT-qPCR)

The protocols and sequences of primers for quantifying HCV RNA, human IFN-β, MxA, ISG56, STING and Actin were described previously [15, 42]. The sequences of primers for quantifying TRIF mRNA were F: 5′- ATCTGGGAGTGTTCGTCCAG-3′; R: 5′- CCAGACTGTGTCATCCCCTT-3′.

### Western blotting

The protocol was as described previously [15]. Antibodies against Flag and β-actin were obtained from Abmart (Shanghai, China). HCV NS3 and core monoclonal antibodies were generated by our laboratory [43]. The monoclonal antibodies against TRIF, caspase8 and caspase9 were obtained from Cell Signaling Technology (Danvers, MA, USA). The anti-MAVS monoclonal antibody, Goat-anti Mouse HRP antibody and Goat-anti Rabbit HRP antibody were obtained from Santa Cruz Biotechnology (Heidelberg, Germany). The protein levels were quantified by Image J from at least two independent experiments, normalized against internal Actin control and expressed as values relative to the control or mock infection. The statistical analysis of protein quantification was shown in S8 Fig.

### STING knockdown assay

The siRNA-based knockdown protocol was as described previously [15]. Briefly, PH5CH8 cells seeded in 12-well plates were transfected with 1 μg of siRNA plasmids for one day, followed by culturing in DMEM containing 3 μg/ml puromycin for another day. After the puromycin selection, the cells were either transfected with 400 ng/well poly(I:C) for 16 h or treated with 50 μg/ml poly(I:C) in culture medium for 6 h. The cells were then analyzed for IFN-β mRNA level by RT-qPCR assay.

### Generation of Huh7-TLR3 cell line

Lentiviruses expressing TLR3 were generated by co-transfecting HEK293T cells with pLVX-TLR3-IRES, packaging vector psPAX2 and envelop vector pMD2.G using Lipofectamine 2000 (Invitrogen). Culture supernatants containing lentiviruses were harvested at 48 h post-transfection, passed through a 0.45 μM-pore-size filter, and used to infect Huh7 cells. Huh7 cells seeded in 6-well plate were infected with 1.2 ml lentivirus expressing TLR3 to generate Huh7-TLR3 stable cell line. The TLR3 expression in Huh7-TLR3 cells was verified by Western blot.

### Generation of Caspase8-, Caspase9- and MAVS-knockout cells by lenti-CRISPR-Cas9

The sequences of 5 sgRNAs targeting caspase9 were #1: 5′-GCAGGCAGCTGATCATAGATC-3′; #2: 5′-GCTTCGTTTCTGCGAACTAAC-3′; #3: 5′-GCTCTTGAGAGTTTGAG-3′; #4: 5′-GCTGAGCATGGAGCCCTG-3′; #5: 5′-GACTCACGGCAGAAGTTC-3′. Four sgRNAs targeting caspase8 were #1: 5′-GCTCAGGAACTTGAGGG-3′; #2: 5′-GAATGTAGTCCAGGCTC-3′; #3: 5′-GCCTGGACTACATTCCGCAA-3′; #4: 5′-GCTCTTCCGAATTAATAGAC-3′. Three sgRNAs targeting MAVS were #1: 5’-GGTTCCCTGAGAGTGTGC- 3’; #2: 5’-GTGAGCTAGTTGATCTCG-3’; #3: 5’-GCACACTCTCAGGGAAC-3’. To generate lentiviruses expressing sgRNA, HEK293T cells seeded at a density of 1x 10^6^ cells per well in 6-well plates a day ago were transfected with 1.3 μg of VSV-G expressing plasmid, 2.5 μg of Pcmv-dR8.91 plasmid and 2.5 μg of lenti-caspase8-sgRNAs or lenti-caspase9-sgRNAs or lenti-MAVS-sgRNAs using Lipofectamine 2000. Culture supernatants containing lentiviruses were harvested at 48 h post-transfection, passed through a 0.45 μM-pore-size filter. HEK293T cells seeded in 6-well plate at a density of 5 x 10^5^ per well were infected by 1.2ml lentiviruses expressing caspase9-sgRNAs to generate HEK293T-sgcaspase9 stable cell line. HEK293T or Huh7-TLR3 cells seeded in 6-well plate at a density of 5 x 10^5^ per well were infected with 1.2ml lentivirus expressing caspase8-sgRNAs to generate HEK293T-sgcaspase8 or Huh7-TLR3-sgcaspase8 stable cell lines. Huh7-TLR3 cells seeded in 6-well plate at a density of 5 x 10^5^ per well were infected with 1.2ml lentivirus expressing MAVS-sgRNAs to generate Huh7-TLR3-sgMAVS stable cell lines. The knockout of caspase9, caspase8 or MAVS in the transduced cells was determined by Western blot. To subclone caspase8-knockout Huh7-TLR3 cells, Huh7-TLR3-sgcaspase8-#1 cells were diluted and plated in 96-well plates with a density of 0.9 cell per well. The cells were grown in complete DMEM supplemented with filtered culture supernatants from Huh7 cell. The knockout was verified by genomic sequencing and Western blot.

### Statistical analysis

Statistical analysis was performed using GraphPad Prism 5 software. Student’s t test was used for analyzing the difference between two groups, and One-way analysis of variance (ANOVA) followed by Tukey post hoc test was used for analyzing the differences among groups of more than three. ns, P>0.05; *P<0.05.

## Supporting information

S1 Fig Kinetics of TRIF mRNA levels during HCV infection. Huh7 cells were infected by HCVcc (MOI = 5) for the indicated time points. The cells were analyzed for the TRIF mRNA abundance by RT-qPCR. The TRIF mRNA level was normalized against cellular Actin mRNA level, and expressed as values relative to the mock infection control of day 1.
(DOC)

S2 Fig Generation of caspase9 and caspase8 knockout HEK293T cells by CRISPR-Cas9. Five caspase9-specific sgRNAs and 4 caspase8-specific sgRNAs were designed and tested in HEK293T cells by lentivirus-based transduction. The protein level of caspase9 (A) or caspase8 (B) was analyzed by immunoblotting with caspase9- or caspase8-specific antibodies.
(DOC)

S3 Fig Generation of Huh7-TLR3 cells. (A) Western blot analysis of TLR3 expression in Huh7-TLR3 cells that were transduced by lentivirus expressing TLR3. The immunoblotting assay was performed using an anti-TLR3 antibody. Huh7-TLR3 cells were treated with poly(I:C) in culture medium (M-pIC) for 6 h, and then analyzed by RT-qPCR to detect the mRNA abundance of IFN-β (B) and MxA (C). Both RNAs were normalized against cellular Actin mRNA level, and expressed as values relative to the mock control. The error bars represent standard deviations from three independent experiments. Student’s t test was used for statistical analysis. ns, P>0.05; *P<0.05.
(DOC)

S4 Fig The MAVS knockout has no effect on the TLR3-mediated IFN signaling. Huh7-TLR3-sgMAVS-#1 cells were treated by poly(I:C) for 6 h and then analyzed by RT-qPCR to detect the mRNA abundance of IFN-β (A), MxA (B) and ISG56 (C). The error bars represent standard deviations from three independent experiments. Student’s t test was used for statistical analysis. ns, P>0.05.
(DOC)

S5 Fig Generation of caspase8 knockout Huh7-TLR3 cells. The caspase8 knockout Huh7-TLR3 cells were generated by the lentiviral vector-based CRISPR-Cas9 system. The protein level of caspase8 was analyzed by immunoblotting with an anti-caspase8 antibody.
(DOC)

S6 Fig Caspase8 knockout restores TRIF expression and enhances IFN signaling during HCV infection. (A) Subcloning of Huh7-TLR3-sgCaspase8-#1 cells. Clone 4 (c4) was selected and analyzed for caspase 8 expression by Western blot using an anti-caspase8 antibody. (B-F) Huh7-TLR3-sgCaspase8-#1c4 cells were infected with HCVcc (MOI = 5) for the indicated time points. The cells were analyzed by immunoblotting using anti-TRIF, anti-Core and anti-Actin antibodies (B) or by RT-qPCR to detect the mRNA abundance of IFN-β (C), MxA (D), ISG56 (E) and HCV (F). The TRIF protein level was quantified by Image J, normalized against internal Actin control and expressed as values relative to the mock infection controls from two independent experiments. The IFN-β, MxA and ISG56 mRNAs were normalized against cellular Actin mRNA level and expressed as values relative to the mock infection control. HCV RNA was expressed as values relative to the Actin mRNA level. The error bars represent standard deviations from three independent experiments. Student’s t test was used for statistical analysis. ns, P>0.05; *P<0.05.
(DOC)

S7 Fig RLR signaling is not involved in the enhancement of IFN activation in HCV-infected Huh7-TLR3 caspase8 knockout cells. (A) Western blot analysis of MAVS protein in Huh7-TLR3-sgCaspase8-#1 cells transduced with sgRNAs targeting MAVS. (B) Huh7-TLR3-sgCaspase8-#1-sgMAVS-#1 cells or control cells were transfected with HCV 3’-UTR RNA or poly(I:C) for 16 h, and then analyzed by RT-qPCR to detect the mRNA abundance of IFN-β. (C-F) Huh7-TLR3-sgCaspase8-#1-sgMAVS-#1 cells as well as control cells were infected by HCVcc (MOI = 5) for the indicated time points. The cells were analyzed by RT-qPCR to detect the mRNA abundance of IFN-β (C), MxA (D), ISG56 (E) and HCV (F). The IFN-β, MxA and ISG56 mRNAs were normalized against cellular Actin mRNA level and expressed as values relative to the mock infection control. HCV RNA was expressed as values relative to the Actin mRNA level. The error bars represent standard deviations from three independent experiments. Student’s t test was used for statistical analysis. ns, P>0.05; *P<0.05.
(DOC)

S8 Fig The statistical analysis of protein quantification western blots of this study. (A) Quantification of FLAG-TRIF protein in Western blot of Fig 3B. (B) Quantification of TRIF protein in Western blot of Fig 3D. (C) Quantification of MAVS protein in Western blot of Fig 3D. (D) Quantification of TRIF protein in Western blot of Fig 3E. (E) Quantification of MAVS protein in Western blot of Fig 3E. (F) Quantification of TRIF protein in Western blot of Fig 3F. (G) Quantification of TRIF protein in Western blot of Fig 4A. (H) Quantification of TRIF protein in Western blot of Fig 4B. (I) Quantification of TRIF protein in Western blot of Fig 4C. (J) Quantification of TRIF protein in Western blot of Fig 4D. (K) Quantification of TRIF protein in Western blot of Fig 5A. (L) Quantification of TRIF protein in Western blot of Fig 7A. (M) Quantification of TRIF protein in Western blot of S5B Fig. All proteins were quantified by Image J, normalized against internal Actin control and expressed as values relative to the vector or mock infection controls from at least two independent experiments. Student’s t test was used for statistical analysis. ns, P>0.05; *P<0.05.
(DOC)
