# Correction: Hepatitis C virus NS4B induces the degradation of TRIF to inhibit TLR3-mediated interferon signaling pathway

**DOI:** 10.1371/journal.ppat.1007455

**Published:** 2018-11-16

**Authors:** Yisha Liang, Xuezhi Cao, Qiang Ding, Yanan Zhao, Zhenliang He, Jin Zhong

In the Funding section, the grant number from the funder National Natural Science Foundation of China is listed incorrectly. The correct grant number is: 81330039.
