# Supplementary material for: Hepatitis C virus NS4B induces the degradation of TRIF to inhibit TLR3-mediated interferon signaling pathway
Source: PLoS Pathog. 2018 May 21;14(5):e1007075. doi: 10.1371/journal.ppat.1007075 (PMC5983870; doi:10.1371/journal.ppat.1007075)
Supplement: S1 Fig — Huh7 cells were infected by HCVcc (MOI = 5) for the indicated time points. The cells were analyzed for the TRIF mRNA abundance by RT-qPCR. The TRIF mRNA level was normalized against cellular Actin mRNA level, and expressed as values relative to the mock infection control of day 1. (DOC) [file ppat.1007075.s001.doc]

S1 Figure


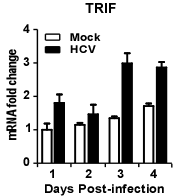


**S1 Fig. Kinetics of TRIF mRNA levels during HCV infection.** Huh7 cells were infected by HCVcc (MOI=5) for the indicated time points. The cells were analyzed for the TRIF mRNA abundance by RT-qPCR. The TRIF mRNA level was normalized against cellular Actin mRNA level, and expressed as values relative to the mock infection control of day 1.
