# Supplementary material for: Hepatitis C virus NS4B induces the degradation of TRIF to inhibit TLR3-mediated interferon signaling pathway
Source: PLoS Pathog. 2018 May 21;14(5):e1007075. doi: 10.1371/journal.ppat.1007075 (PMC5983870; doi:10.1371/journal.ppat.1007075)
Supplement: S2 Fig — Five caspase9-specific sgRNAs and 4 caspase8-specific sgRNAs were designed and tested in HEK293T cells by lentivirus-based transduction. The protein level of caspase9 (A) or caspase8 (B) was analyzed by immunoblotting with caspase9- or caspase8-specific antibodies. (DOC) [file ppat.1007075.s002.doc]

S2 Figure

**S2 Fig. Generation of caspase9 and caspase8 knockout HEK293T cells by CRISPR-Cas9.** Five caspase9-specific sgRNAs and 4 caspase8-specific sgRNAs were designed and tested in HEK293T cells by lentivirus-based transduction. The protein level of caspase9 (A) or caspase8 (B) was analyzed by immunoblotting with caspase9- or caspase8-specific antibodies.
