# Supplementary material for: Hepatitis C virus NS4B induces the degradation of TRIF to inhibit TLR3-mediated interferon signaling pathway
Source: PLoS Pathog. 2018 May 21;14(5):e1007075. doi: 10.1371/journal.ppat.1007075 (PMC5983870; doi:10.1371/journal.ppat.1007075)
Supplement: S3 Fig — (A) Western blot analysis of TLR3 expression in Huh7-TLR3 cells that were transduced by lentivirus expressing TLR3. The immunoblotting assay was performed using an anti-TLR3 antibody. Huh7-TLR3 cells were treated with poly(I:C) in culture medium (M-pIC) for 6 h, and then analyzed by RT-qPCR to detect the mRNA abundance of IFN-β (B) and MxA (C). Both RNAs were normalized against cellular Actin mRNA level, and expressed as values relative to the mock control. The error bars represent standard deviations from three independent experiments. Student’s t test was used for statistical analysis. ns, P>0.05; *P<0.05. (DOC) [file ppat.1007075.s003.doc]

S3 Figure

**S3 Fig. Generation of Huh7-TLR3 cells.** (A) Western blot analysis of TLR3 expression in Huh7-TLR3 cells that were transduced by lentivirus expressing TLR3. The immunoblotting assay was performed using an anti-TLR3 antibody. Huh7-TLR3 cells were treated with poly(I:C) in culture medium (M-pIC) for 6 h, and then analyzed by RT-qPCR to detect the mRNA abundance of IFN-β (B) and MxA (C). Both RNAs were normalized against cellular Actin mRNA level, and expressed as values relative to the mock control. The error bars represent standard deviations from three independent experiments. Student’s t test was used for statistical analysis. ns, P>0.05; *P<0.05.
