# Supplementary material for: Hepatitis C virus NS4B induces the degradation of TRIF to inhibit TLR3-mediated interferon signaling pathway
Source: PLoS Pathog. 2018 May 21;14(5):e1007075. doi: 10.1371/journal.ppat.1007075 (PMC5983870; doi:10.1371/journal.ppat.1007075)
Supplement: S4 Fig — Huh7-TLR3-sgMAVS-#1 cells were treated by poly(I:C) for 6 h and then analyzed by RT-qPCR to detect the mRNA abundance of IFN-β (A), MxA (B) and ISG56 (C). The error bars represent standard deviations from three independent experiments. Student’s t test was used for statistical analysis. ns, P>0.05. (DOC) [file ppat.1007075.s004.doc]

S4 Figure


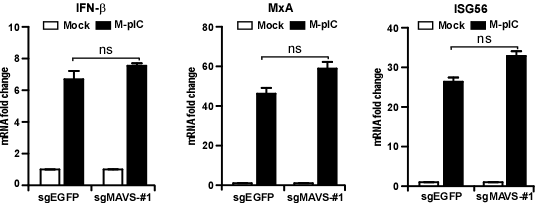


**S4 Fig. The MAVS knockout has no effect on the TLR3-mediated IFN signaling.** Huh7-TLR3-sgMAVS-#1 cells were treated by poly(I:C) for 6 h and then analyzed by RT-qPCR to detect the mRNA abundance of of IFN-β (A), MxA (B) and ISG56 (C). The error bars represent standard deviations from three independent experiments. Student’s t test was used for statistical analysis. ns, P>0.05.
