# Supplementary material for: Hepatitis C virus NS4B induces the degradation of TRIF to inhibit TLR3-mediated interferon signaling pathway
Source: PLoS Pathog. 2018 May 21;14(5):e1007075. doi: 10.1371/journal.ppat.1007075 (PMC5983870; doi:10.1371/journal.ppat.1007075)
Supplement: S5 Fig — The caspase8 knockout Huh7-TLR3 cells were generated by the lentiviral vector-based CRISPR-Cas9 system. The protein level of caspase8 was analyzed by immunoblotting with an anti-caspase8 antibody. (DOC) [file ppat.1007075.s005.doc]

S5 Figure

**S5 Fig. Generation of caspase8 knockout Huh7-TLR3 cells.** The caspase8 knockout Huh7-TLR3 cells were generated by the lentiviral vector-based CRISPR-Cas9 system. The protein level of caspase8 was analyzed by immunoblotting with an anti-caspase8 antibody.
