# Supplementary material for: Hepatitis C virus NS4B induces the degradation of TRIF to inhibit TLR3-mediated interferon signaling pathway
Source: PLoS Pathog. 2018 May 21;14(5):e1007075. doi: 10.1371/journal.ppat.1007075 (PMC5983870; doi:10.1371/journal.ppat.1007075)
Supplement: S6 Fig — (A) Subcloning of Huh7-TLR3-sgCaspase8-#1 cells. Clone 4 (c4) was selected and analyzed for caspase 8 expression by Western blot using an anti-caspase8 antibody. (B-F) Huh7-TLR3-sgCaspase8-#1c4 cells were infected with HCVcc (MOI = 5) for the indicated time points. The cells were analyzed by immunoblotting using anti-TRIF, anti-Core and anti-Actin antibodies (B) or by RT-qPCR to detect the mRNA abundance of IFN-β (C), MxA (D), ISG56 (E) and HCV (F). The TRIF protein level was quantified by Image J, normalized against internal Actin control and expressed as values relative to the mock infection controls from two independent experiments. The IFN-β, MxA and ISG56 mRNAs were normalized against cellular Actin mRNA level and expressed as values relative to the mock infection control. HCV RNA was expressed as values relative to the Actin mRNA level. The error bars represent standard deviations from three independent experiments. Student’s t test was used for statistical analysis. ns, P>0.05; *P<0.05. (DOC) [file ppat.1007075.s006.doc]

S6 Figure


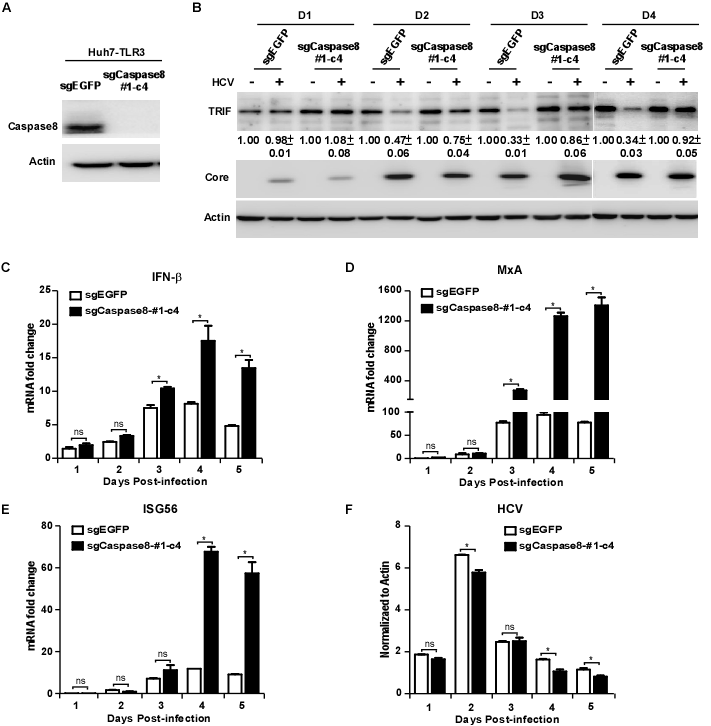


**S6 Fig. Caspase8 knockout restores TRIF expression and enhances IFN signaling during HCV infection.** (A) Subcloning of Huh7-TLR3-sgCaspase8-#1 cells. Clone 4 (c4) was selected and analyzed for caspase 8 expression by Western blot using an anti-caspase8 antibody. (B-F) Huh7-TLR3-sgCaspase8-#1c4 cells were infected with HCVcc (MOI=5) for the indicated time points. The cells were analyzed by immunoblotting using anti-TRIF, anti-Core and anti-Actin antibodies (B) or by RT-qPCR to detect the mRNA abundance of IFN-β (C), MxA (D), ISG56 (E) and HCV (F). The TRIF protein level was quantified by Image J, normalized against internal Actin control and expressed as values relative to the mock infection controls from two independent experiments. The IFN-β, MxA and ISG56 mRNAs were normalized against cellular Actin mRNA level and expressed as values relative to the mock infection control. HCV RNA was expressed as values relative to the Actin mRNA level. The error bars represent standard deviations from three independent experiments. Student’s t test was used for statistical analysis. ns, P>0.05; *P<0.05.
