# Supplementary material for: Hepatitis C virus NS4B induces the degradation of TRIF to inhibit TLR3-mediated interferon signaling pathway
Source: PLoS Pathog. 2018 May 21;14(5):e1007075. doi: 10.1371/journal.ppat.1007075 (PMC5983870; doi:10.1371/journal.ppat.1007075)
Supplement: S7 Fig — (A) Western blot analysis of MAVS protein in Huh7-TLR3-sgCaspase8-#1 cells transduced with sgRNAs targeting MAVS. (B) Huh7-TLR3-sgCaspase8-#1-sgMAVS-#1 cells or control cells were transfected with HCV 3’-UTR RNA or poly(I:C) for 16 h, and then analyzed by RT-qPCR to detect the mRNA abundance of IFN-β. (C-F) Huh7-TLR3-sgCaspase8-#1-sgMAVS-#1 cells as well as control cells were infected by HCVcc (MOI = 5) for the indicated time points. The cells were analyzed by RT-qPCR to detect the mRNA abundance of IFN-β (C), MxA (D), ISG56 (E) and HCV (F). The IFN-β, MxA and ISG56 mRNAs were normalized against cellular Actin mRNA level and expressed as values relative to the mock infection control. HCV RNA was expressed as values relative to the Actin mRNA level. The error bars represent standard deviations from three independent experiments. Student’s t test was used for statistical analysis. ns, P>0.05; *P<0.05. (DOC) [file ppat.1007075.s007.doc]

S7 Figure


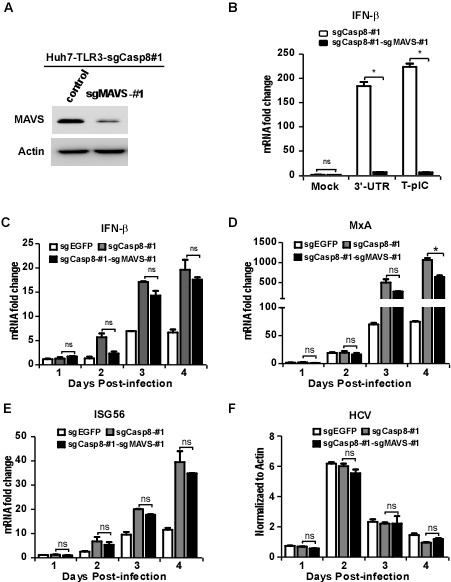


**S7 Fig. RLR signaling is not involved in the enhancement of IFN activation in HCV-infected Huh7-TLR3 caspase8 knockout cells.** (A) Western blot analysis of MAVS protein in Huh7-TLR3-sgCaspase8-#1 cells transduced with sgRNAs targeting MAVS. (B) Huh7-TLR3-sgCaspase8-#1-sgMAVS-#1 cells or control cells were transfected with HCV 3’-UTR RNA or poly(I:C) for 16 h, and then analyzed by RT-qPCR to detect the mRNA abundance of IFN- β. (C-F) Huh7-TLR3-sgCaspase8-#1-sgMAVS-#1 cells as well as control cells were infected by HCVcc (MOI=5) for the indicated time points. The cells were analyzed by RT-qPCR to detect the mRNA abundance of IFN-β (C), MxA (D), ISG56 (E) and HCV (F). The IFN-β, MxA and ISG56 mRNAs were normalized against cellular Actin mRNA level and expressed as values relative to the mock infection control. HCV RNA was expressed as values relative to the Actin mRNA level. The error bars represent standard deviations from three independent experiments. Student’s t test was used for statistical analysis. ns, P>0.05; *P<0.05.
