# Supplementary material for: Hepatitis C virus NS4B induces the degradation of TRIF to inhibit TLR3-mediated interferon signaling pathway
Source: PLoS Pathog. 2018 May 21;14(5):e1007075. doi: 10.1371/journal.ppat.1007075 (PMC5983870; doi:10.1371/journal.ppat.1007075)
Supplement: S8 Fig — (A) Quantification of FLAG-TRIF protein in Western blot of Fig 3B. (B) Quantification of TRIF protein in Western blot of Fig 3D. (C) Quantification of MAVS protein in Western blot of Fig 3D. (D) Quantification of TRIF protein in Western blot of Fig 3E. (E) Quantification of MAVS protein in Western blot of Fig 3E. (F) Quantification of TRIF protein in Western blot of Fig 3F. (G) Quantification of TRIF protein in Western blot of Fig 4A. (H) Quantification of TRIF protein in Western blot of Fig 4B. (I) Quantification of TRIF protein in Western blot of Fig 4C. (J) Quantification of TRIF protein in Western blot of Fig 4D. (K) Quantification of TRIF protein in Western blot of Fig 5A. (L) Quantification of TRIF protein in Western blot of Fig 7A. (M) Quantification of TRIF protein in Western blot of S5B Fig. All proteins were quantified by Image J, normalized against internal Actin control and expressed as values relative to the vector or mock infection controls from at least two independent experiments. Student’s t test was used for statistical analysis. ns, P>0.05; *P<0.05. (DOC) [file ppat.1007075.s008.doc]

S8 Figure


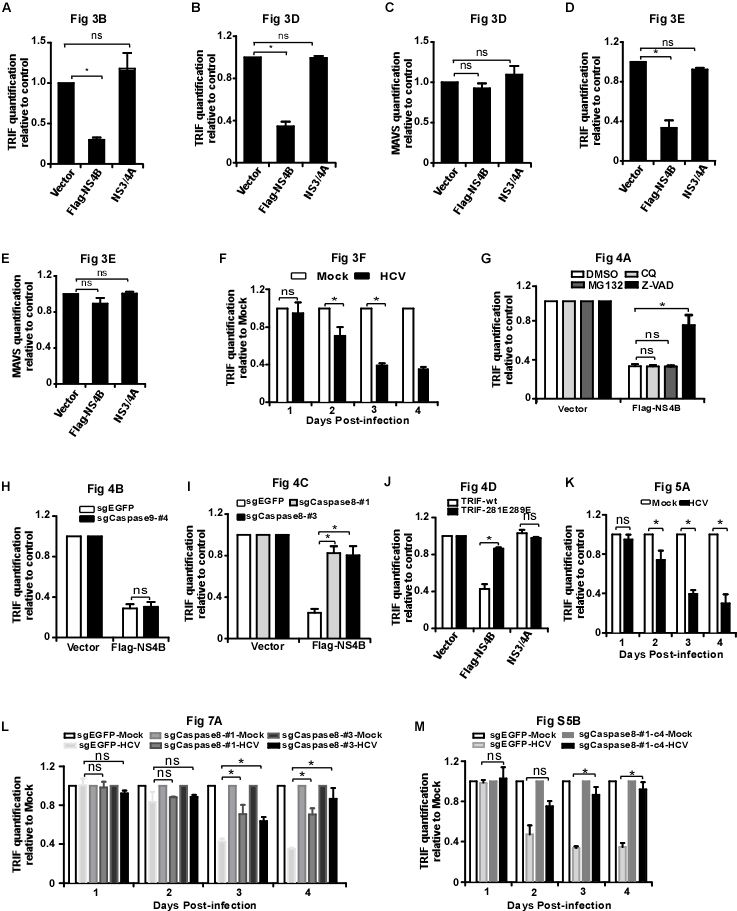


**S8 Fig. The statistical analysis of protein quantification Western blots of this study.** (A) Quantification of FLAG-TRIF protein in Western blot of Fig 3B. (B) Quantification of TRIF protein in Western blot of Fig 3D. (C) Quantification of MAVS protein in Western blot of Fig 3D. (D) Quantification of TRIF protein in Western blot of Fig 3E. (E) Quantification of MAVS protein in Western blot of Fig 3E. (F) Quantification of TRIF protein in Western blot of Fig 3F. (G) Quantification of TRIF protein in Western blot of Fig 4A. (H) Quantification of TRIF protein in Western blot of Fig 4B. (I) Quantification of TRIF protein in Western blot of Fig 4C. (J) Quantification of TRIF protein in Western blot of Fig 4D. (K) Quantification of TRIF protein in Western blot of Fig 5A. (L) Quantification of TRIF protein in Western blot of Fig 7A. (M) Quantification of TRIF protein in Western blot of Fig S5B. All proteins were quantified by Image J, normalized against internal Actin control and expressed as values relative to the vector or mock infection controls from at least two independent experiments. Student’s t test was used for statistical analysis. ns, P>0.05; *P<0.05.
